# Novel Non-Invasive Radiomic Signature on CT Scans Predicts Response to Platinum-Based Chemotherapy and Is Prognostic of Overall Survival in Small Cell Lung Cancer

**DOI:** 10.3389/fonc.2021.744724

**Published:** 2021-10-20

**Authors:** Prantesh Jain, Mohammadhadi Khorrami, Amit Gupta, Prabhakar Rajiah, Kaustav Bera, Vidya Sankar Viswanathan, Pingfu Fu, Afshin Dowlati, Anant Madabhushi

**Affiliations:** ^1^ Department of Hematology and Oncology, University Hospitals Cleveland Medical Center, Cleveland, OH, United States; ^2^ Department of Biomedical Engineering, Case Western Reserve University, Cleveland, OH, United States; ^3^ Department of Radiology, University Hospitals Cleveland Medical Center, Cleveland, OH, United States; ^4^ Department of Radiology, Mayo Clinic Minnesota, Rochester, MN, United States; ^5^ Department of Population and Quantitative Health Sciences, Case Western Reserve University (CWRU), Cleveland, OH, United States; ^6^ Louis Stokes Cleveland Veterans Administration Medical Center, Cleveland, OH, United States

**Keywords:** radiomics, computed tomography, small cell lung cancer (SCLC), chemotherapy, overall survival, progression-free survival

## Abstract

**Background:**

Small cell lung cancer (SCLC) is an aggressive malignancy characterized by initial chemosensitivity followed by resistance and rapid progression. Presently, there are no predictive biomarkers that can accurately guide the use of systemic therapy in SCLC patients. This study explores the role of radiomic features from both within and around the tumor lesion on pretreatment CT scans to a) prognosticate overall survival (OS) and b) predict response to chemotherapy.

**Methods:**

One hundred fifty-three SCLC patients who had received chemotherapy were included. Lung tumors were contoured by an expert reader. The patients were divided randomly into approximately equally sized training (S^tr^ = 77) and test sets (S^te^ = 76). Textural descriptors were extracted from the nodule (intratumoral) and parenchymal regions surrounding the nodule (peritumoral). The clinical endpoints of this study were OS, progression-free survival (PFS), and best objective response to chemotherapy. Patients with complete or partial response were defined as “responders,” and those with stable or progression of disease were defined as “non-responders.” The radiomic risk score (RRS) was generated by using the least absolute shrinkage and selection operator (LASSO) with the Cox regression model. Patients were classified into the high-risk or low-risk groups based on the median of RRS. Association of the radiomic signature with OS was evaluated on S^tr^ and then tested on S^te^. The features identified by LASSO were then used to train a linear discriminant analysis (LDA) classifier (M^Rad^) to predict response to chemotherapy. A prognostic nomogram (N^Rad+Clin^) was also developed on S^tr^ by combining clinical and prognostic radiomic features and validated on S^te^. The Kaplan–Meier survival analysis and log-rank statistical tests were performed to assess the discriminative ability of the features. The discrimination performance of the N^Rad+Clin^ was assessed by Harrell’s C-index. To estimate the clinical utility of the nomogram, decision curve analysis (DCA) was performed by calculating the net benefits for a range of threshold probabilities in predicting which high-risk patients should receive more aggressive treatment as compared with the low-risk patients.

**Results:**

A univariable Cox regression analysis indicated that RRS was significantly associated with OS in S^tr^ (HR: 1.53; 95% CI, [1.1–2.2; p = 0.021]; C-index = 0.72) and S^te^ (HR: 1.4, [1.1–1.82], p = 0.0127; C-index = 0.69). The RRS was also significantly associated with PFS in S^tr^ (HR: 1.89, [1.4–4.61], p = 0.047; C-index = 0.7) and S^te^ (HR: 1.641, [1.1–2.77], p = 0.04; C-index = 0.67). M^Rad^ was able to predict response to chemotherapy with an area under the receiver operating characteristic curve (AUC) of 0.76 ± 0.03 within S^tr^ and 0.72 within S^te^. Predictors, including the RRS, gender, age, stage, and smoking status, were used in the prognostic nomogram. The discrimination ability of the N^Rad+Clin^ model on S^tr^ and S^te^ was C-index [95% CI]: 0.68 [0.66–0.71] and 0.67 [0.63–0.69], respectively. DCA indicated that the N^Rad+Clin^ model was clinically useful.

**Conclusions:**

Radiomic features extracted within and around the lung tumor on CT images were both prognostic of OS and predictive of response to chemotherapy in SCLC patients.

## 1 Introduction

Lung cancer remains the leading cause of cancer-related mortality worldwide. Traditionally, primary lung cancers have been divided into non-small cell lung cancer (NSCLC) and small cell lung cancer (SCLC), with majority of them (85%) being of the NSCLC subtype (1). SCLC is an aggressive neuroendocrine (NE) malignancy that accounts for 13% to 15% of all lung cancers and is strongly associated with smoking. Besides Tumor-Node-Metastasis (TNM) staging, SCLC can be classified into limited-stage disease (LS-SCLC—tumor confined to single radiation port with or without loco-regional adenopathy) and extensive-stage disease (ES-SCLC—tumor not confined to single radiation port) (2). This two-stage system has therapeutic and prognostic implications with 5-year relative survival rates of 31% for LS-SCLC and 2% for ES-SCLC (3).

The current treatment modalities for SCLC include systemic chemotherapy, immunotherapy, thoracic radiation, and prophylactic cranial irradiation, depending on the tumor stage. While SCLC is very responsive to initial treatment, most patients develop early resistance to conventional therapies, show rapid progression, and relapse with decreased sensitivity to further pharmacological treatment (4, 5); and fewer than 10% patients enjoy long-term survival (6–8). There has been little improvement in outcome over the past few decades with the addition of immunotherapy to chemotherapy in an unselected patient population, and thus, platinum-based chemotherapy remains the mainstay of systemic treatment (9). While younger age, good performance status (PS), normal creatinine level, and normal lactate dehydrogenase (LDH) are favorable prognostic factors (10), there are no reliable predictive biomarkers that can identify SCLC patients who will benefit from cytotoxic chemotherapy or patients at a high risk of relapse, although recently it has been shown that patients with wild-type retinoblastoma gene are chemotherapy-refractory (11).

CT is a routinely used clinical diagnostic tool for tumor staging and monitoring treatment response. The common presentation of SCLC on a CT scan is a centrally located large parenchymal mass or a mediastinal mass involving at least one hilum. In recent years, computational imaging approaches such as “radiomics” (12) can provide a more detailed feature-based characterization of the disease than possible by visual examination. Radiomics-based biomarkers have been shown to be prognostically useful in different types of therapies for various cancers, including NSCLC (13–16). However, there are no data regarding the role of radiomics in predicting response to chemotherapy or prognosticating outcome for SCLC.

In this study, we sought to identify chemotherapy response as well as prognostic biomarkers for overall survival (OS) and progression-free survival (PFS) in SCLC patients treated with chemotherapy by interrogating the tumor and tumor microenvironment on CT imaging. We hypothesize that quantitative subvisual phenotypic differences in SCLC tumors on CT imaging can be characterized non-invasively to develop predictive and prognostic biomarker signatures to improve decision support in SCLC treatment. With the use of a cohort of 153 patients with SCLC, 77 were used for training the classifier and constructing a radiomic risk score (RRS), whereas the remaining 76 were used for the test set. The choice of an equal split of training and test sets has been employed in previous approaches involving radiomics (14, 15, 17). In addition, a novel prognostic nomogram was constructed by integrating RRS and clinical biomarkers, and its performance for predicting high-risk patients was evaluated by the decision curve analysis (DCA) model.

## 2 Materials and Methods

### 2.1 Datasets and Patient Selection

**Figure 1:**
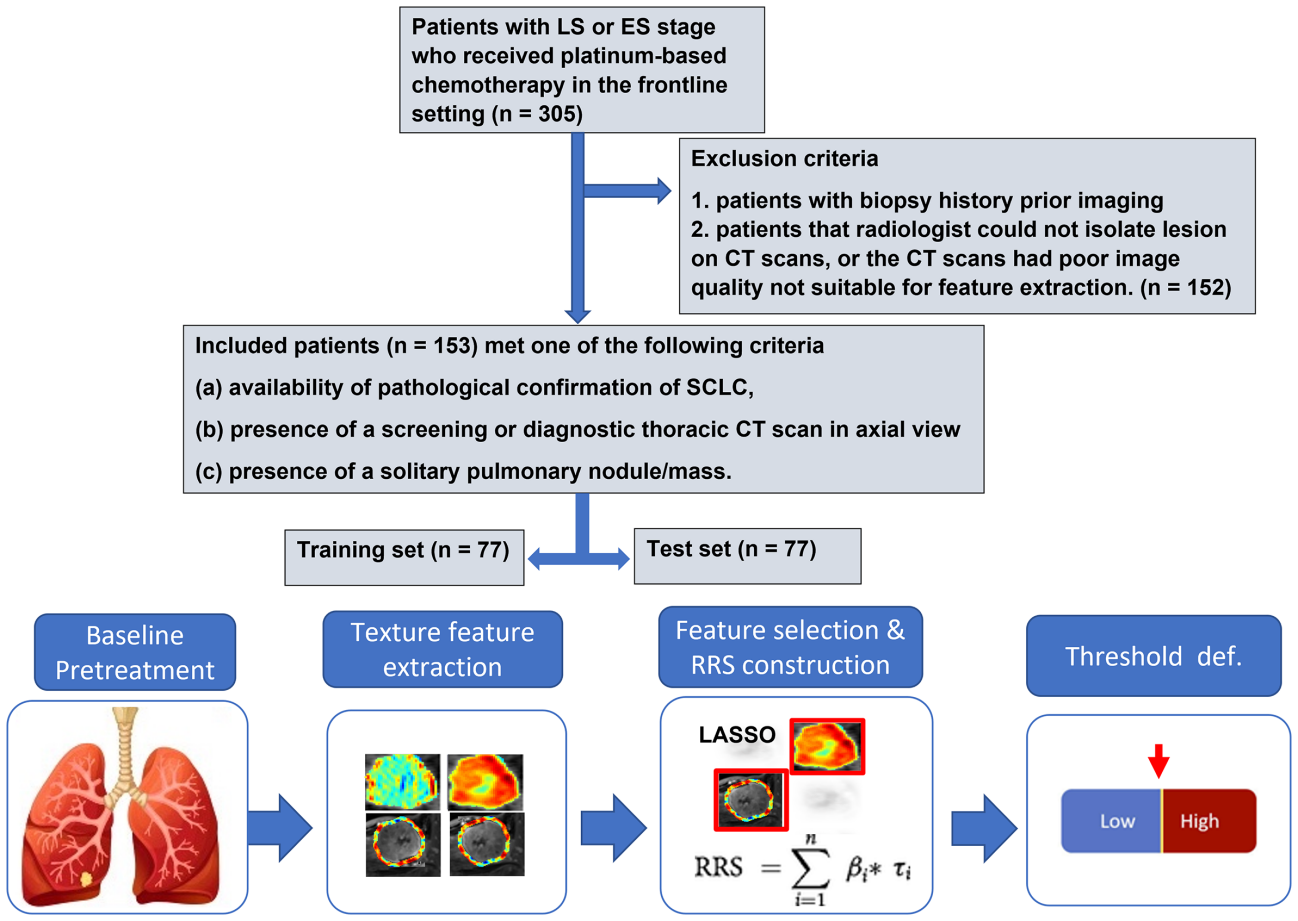
Patient selection and overall experimental design for this study.

This study was conducted in full accordance with the Health Insurance Portability and Accountability Act (HIPAA) regulations after approval from the Institutional Review Board (IRB) at Case Western Reserve University (Cleveland, OH), and the IRB waived the requirements for informed consent of all patients because of the retrospective, non-interventional, and non-therapeutic nature of this study. A total of 305 consecutive patients with LS-SCLC or ES-SCLC treated with platinum-based chemotherapy from April 2004 to March 2018 in University Hospitals Cleveland Medical Center (UHCMC) were identified. All patients that met the following criteria were included: a) availability of pathological confirmation of SCLC, b) presence of diagnostic thoracic CT scan in axial view, and c) presence of a solitary pulmonary nodule/mass. To this cohort of 180 patients, the exclusion criteria were applied to remove scans with CT artifacts and poor image quality not suitable for feature extraction. The final cohort had 153 patients. The training set imbalance has been discussed extensively as an issue in training machine classifiers. To reduce the possible impact of imbalanced data on the machine classifier, the patients were randomly divided into an equal number for training set (S^tr^) that consisted of 77 patients (age: 39–87 years) and a test set (St^e^) of 76 patients (age: 47–90 years). Patient selection and overall experimental design for this study are shown in **Figure 1**.

The non-contrast CT scan images were acquired from all participants at baseline before initiation of chemotherapy from Siemens, GE Medical Systems, Philips, or Toshiba machine utilizing a tube voltage of 100 to 120 kVp. The median time between cancer diagnosis and CT scan acquisition was 10.4 days, and that between diagnosis and first-line therapy was 33.5 days (range: 2–220 days). The dataset also had images acquired from multiple reconstruction kernels. It is known that the different slice thickness and reconstruction kernels of the CT image acquisition affect radiomic feature expression and measurements (18). Therefore, precaution was taken to sample cases from both S^tr^ and S^te^. The slice thickness ranged from 1 to 5 mm (mean = 2.82 mm, SD = 0.71 mm), and the pixel sizes ranged from 0.42 × 0.42 mm to 0.97 × 0.97 mm with an average size of 0.73 × 0.73 mm. **Table 1** shows CT acquisition parameters involving all scanner types, slice thicknesses, kVp, and reconstruction kernels for both the training and test sets.

**Table 1:** CT image acquisition parameter distribution over the training set and test set.

	Training set	Test set
***Patients***	77	76
***Scanner***		
* GE*	6	4
* Siemens*	43	45
* Philips*	23	21
* Toshiba*	5	6
***Thickness***		
1	1	2
1.5	2	0
2	10	12
2.5	0	2
3	10	11
3.2	2	0
5	42	49
***Kernel***		
***GE Med***		
* Standard*	6	4
***Siemens***		
* B20f*	1	2
* B30f*	0	0
* B35f*	5	7
* B35s*	0	0
* B40f*	13	10
* B41f*	0	0
* B50f*	10	7
* B60f*	3	1
* B70f*	14	18
***Philips***		
* A*	0	0
* B*	0	0
* C*	11	8
* D*	12	13
* E*	0	0
* L*	0	0
***Toshiba***		
* FC02*	0	0
* FC08*	5	6
* FC10*	0	0
***kVp***		
* 100*	34	31
* 110*	23	27
* 120*	20	18

### 2.2 Clinical Endpoints

The primary endpoints of this study were response to chemotherapy, OS, and PFS. The OS was measured from the date of diagnosis to the date of death and censored at the date of last follow-up for survivors. PFS was defined as the length of time during the treatment that a patient survives with cancer but without evidence of disease progression or death, whichever occurred earlier. The objective response to chemotherapy was evaluated based on RECIST 1.1. The target lesions were evaluated to assess response, and the following definitions were used: complete response (CR), i.e., the disappearance of all the lesions; partial response (PR), i.e., ≥30% decrease in the sum of the longest diameters of target lesions compared with baseline; progressive disease (PD), i.e., at least 20% increase in the sum of the longest diameters of target lesions with an absolute increase of ≥5 mm; and stable disease (SD), i.e., neither PR nor PD (19). For our study, patients with CR or PR were classified as “responders,” and those with SD or PD were classified as “non-responders.”

### 2.3 Tumor Segmentation

The tumor was identified by a two-board-certified cardiothoracic radiologist (PR) with 20 years of experience and Reader 2 (VV, a physician with 2 years of experience in cardiothoracic radiology research), blinded to each other; and the region of interest (ROI) was manually segmented across all the 2D CT slices of the nodule *via* a hand-annotation tool in axial view on 3D-Slicer software. The radiologist was blinded to clinical data and given the option to vary the window and level setting within the software to efficiently annotate the nodule. The segmented nodules were used to extract the intratumoral texture and shape features. The intratumoral mask was then dilated out to a 20-mm peritumoral radius. The definition of the extent of the peritumoral zone was based on a previous publication (20), which showed that patients with SCLC exhibit a tendency to develop peritumoral edema to a region of 20-mm extent around the tumor. Peritumoral masks were inspected manually and adjusted to include only lung tissue when masks extended into chest wall soft tissues.

### 2.4 Radiomic Feature Extraction

For a given tumor, 2D manual segmentations were assessed in a slice-by-slice basis to pick all representative slices that had the tumor. Since there was an unequal slice spacing (ranging from 1 to 5 mm), 2D radiomic analysis provides for a more consistent approach as compared with 3D analysis, which in turn would have been impacted by the non-uniform z-axis slice spacing. From these slices, 2D texture features were extracted from the intratumoral and peritumoral regions on a per-pixel basis. Within the intratumoral and peritumoral regions, a total of 99 2D radiomic texture descriptors were extracted. These descriptors consisted of features that were selected to capture textural structure of intra- and peritumoral regions. In this study, we extracted 13 Haralick features from gray-level co-occurrence matrix (GLCM) that can extract textural pattern and show variation in tumor microarchitecture, heterogeneity, and local appearance of nodules. In addition, we extracted 25 Laws, 25 Laws–Laplacian, and 48 Gabor features from intra- and peritumoral regions. Laws and Laws–Laplacian are filter-based descriptors that capture combinations of five textural patterns, such as levels (L), edges (E), spots (S), waves (W), and/or ripples (R). The Gabor filter bank was used to capture texture responses at six different spatial frequencies (f = 0, 2, 4, 8, 16, or 32) within the image at eight directional orientations (θ = 0, π/8, π/4, 3π/8, π/2, 5π/8, 3π/4, and 7π/8). A total of 24 shape features were also automatically extracted from the annotated nodules and investigated in the study. Shape features are used to describe the 3D geometrical composition of the segmented nodule structure including size (volume and diameter) and shape measures (sphericity, compactness, and radial distance). First-order statistics (mean, median, standard deviation, skewness, and kurtosis) for each descriptor were computed within the tumor and peritumoral region, resulting in 495 statistical features per region. Each feature was normalized to a mean of zero and standard deviation of 1 across patients before feature selection. All shape and texture features were extracted using an in-house software that was developed on MATLAB 2018 platform (MathWorks Inc., Natick, MA), an approach very similar to implementations of radiomic features like CERR and PyRadiomics (21, 22). To mitigate the effect of different acquisition parameters, only features that were stable in the context of the test–retest RIDER lung CT dataset (23) were selected for more analysis.

### 2.5 Statistical Analysis

#### 2.5.1 Feature Selection

To avoid overfitting due to high complexity of features, least absolute shrinkage and selection operator (LASSO) Cox regression model was used to identify the most prognostic features to OS from highly stable features in S^tr^. LASSO iteratively shrinks the feature coefficient estimates toward zero and involves identification of an optimal tuning parameter lambda (λ) *via* a 100 cross-validation setup. The process that is only run on the training set allows for identification of only those features with non-zero coefficients.

#### 2.5.2 Radiomic Risk Score Generation

The RRS signature was built based on linear combination of non-zero coefficients-selected features. The association of the RRS with OS was first assessed in S^tr^ and then validated in S^te^ by using the Kaplan–Meier survival analysis. According to the rad-score threshold identified by the median of RRS, patients were classified into the high-risk or low-risk categories.

#### 2.5.3 Prognostic Analysis

OS and PFS for univariate analysis were estimated by the Kaplan–Meier method. The multivariable survival analysis with RRS and clinicopathologic biomarker was also employed. A DCA was also used to determine the clinical utility of the RRS in predicting OS by evaluating the net benefit of high-risk patients receiving treatment at different threshold probabilities (24). Net benefit was defined as the summation of benefits minus loss results (false-positive findings) weighted by a factor related to the relative harm of not identifying a high-risk patient who might have low OS versus the relative harm of subjecting a lower-risk patient to more aggressive therapy, when the more intense therapy was not needed.

In addition, a nomogram (N^Rad+Clin^) was constructed as an individualized OS prediction model in S^tr^. Predictors, including the RRS and clinical parameters like gender, age, stage, and smoking status, were added to the prognostic nomogram. The prognostic performance of N^Rad+Clin^ was estimated in S^tr^ and then evaluated in S^te^. The consistency between the actual and predicted probabilities survival was evaluated by a calibration plot.

#### 2.5.4 Classification

A linear discriminant analysis (LDA) classifier (M^Rad^) was trained with the same set of features identified by LASSO. Within S^tr^, the LDA classifier was trained over 100 iterations of threefold cross-validation. The classifier was finally locked down and then evaluated for prediction of response on S^te^. The ability to identify response was primarily assessed by an area under the receiver operating characteristic (ROC) curve (AUC).

#### 2.5.5 Implementation and Statistical Test

The “glmnet” package in R was used for executing the LASSO algorithm. Analysis of OS and PFS outcomes was utilized with survival methods, primarily Cox regression. The model was included indicators for the categorical low- and high-risk OS from RRS along with clinical factors. The survival probabilities of patients classified as low or high risk based on RRS was estimated and illustrated by the Kaplan–Meier curves, and relative HRs with 95% CIs were calculated using the Wald test and the G-rho rank test, in R, version 3.6.3. Survival differences were compared by the log-rank test. A multivariable Cox PH model was used to investigate the independent prognostic effect of the RRS model in comparison with clinical variables, and the likelihood ratio test was applied to confirm the independent prognostic effect of each variable.

The final selection of the model for the nomogram was conducted using a backward step-down selection process based on the Akaike information criterion (25), while internal validation was conducted through 1,000 bootstrap resamples. Harrell’s concordance index (C-index), a quantitative measurement of the performance of the nomogram, was used to assess the discriminative ability of the model in survival analysis. The nomogram and calibration plots were constructed using the “rms” and “SvyNom” packages.

A heatmap dendrogram was used to display unsupervised hierarchal clustering using the radiomic texture features. A consensus clustering approach was also used to determine the number and affiliation of possible clusters within all the patient studies. Nodules belonging to different clusters may have minimal correlation, while nodules within a cluster are likely to have a high intra-class correlation.

Accuracy, precision, sensitivity, specificity, and kappa agreement between the predicted response of classifier and actual response were also computed at the optimal operating point of the ROC curve, while the operating point was defined as the threshold that maximized overall accuracy.

Differences between clinical categories were assessed using Fisher’s exact test, while a two-sided Wilcoxon test was used for continuous variables. For continuous variables, the interquartile range (IQR), a measure of statistical dispersion, was also reported. IQR was calculated as the difference between the 75th and 25th percentiles.

## 3 Results

### 3.1 Patient Analysis

A total of 153 patients with SCLC were included for analysis with a median age of 66 years (34−90), with 72.8% men, median OS of 9.37, and median PFS of 8.35 months. All patients with LS or ES were treated with platinum-based chemotherapy. Of these, 86% patients had extensive-stage disease, and 14% were of limited stage. No statistically significant difference was found in baseline clinical characteristics between responders and non-responders, chemotherapy agents (carboplatin and cisplatin), or time between CT acquisition and first-line chemotherapy. Chemotherapy response was achieved in 100 (66%) patients, who were labeled as responders (R), and the remaining 53 (34%) were labeled as non-responders (NR). All clinical characteristics are listed in **Table 2**.

**Table 2:** Demographics and clinical characteristics for patients, categorized by training and test sets.

Characteristics		All patients (n = 153)	Training set (n = 77)	Test set (n = 76)	p-Value
**Sex**	Male	78 (52%)	43 (56%)	35 (46%)	0.26
	Female	75 (48%)	34 (44%)	41 (54%)	
**Age**	Median (IQR) [95% CI]	66 (13) [64.97–68.16]	6712.5) [64.00–68.67]	66 (14) [64.62–68.98]	0.89
**Race**	White	98 (82%)	48 (62%)	50 (66%)	0.73
	Black	55 (18%)	29 (38%)	26 (34%)	
**Smoking**	Never	6 (4%)	3 (4%)	3 (4%)	1.00
	Former/current	147 (96%)	74 (96%)	73 (96%)	
**Stage**	Limited stage	21 (14%)	10 (13%)	11 (14%)	0.81
	Extensive stage	132 (86%)	67 (87%)	65 (86%)	
**Chemotherapy Agents**	Carboplatin	64 (42%)	30 (39%)	34 (45%)	0.51
	Cisplatin	89 (58%)	47 (61%)	42 (55%)	
**Median OS**	Months (IQR) [95% CI]	9.37 (12.73) [11.63–15.77]	8.27 (12.55) [10.10–16.40]	10.18 (12.48) [11.46–16.85]	0.12
**Median PFS**	Months (IQR) [95% CI]	8.35 (11.8) [11.2–16.84]	7.57 (12.2) [9.68–17.36]	9.23 (11.9) [10.37–18.68]	0.19

### 3.2 Radiomic Features From Pretreatment CT Scans Were Associated With Overall Survival in Small Cell Lung Cancer

**Figure 2:**
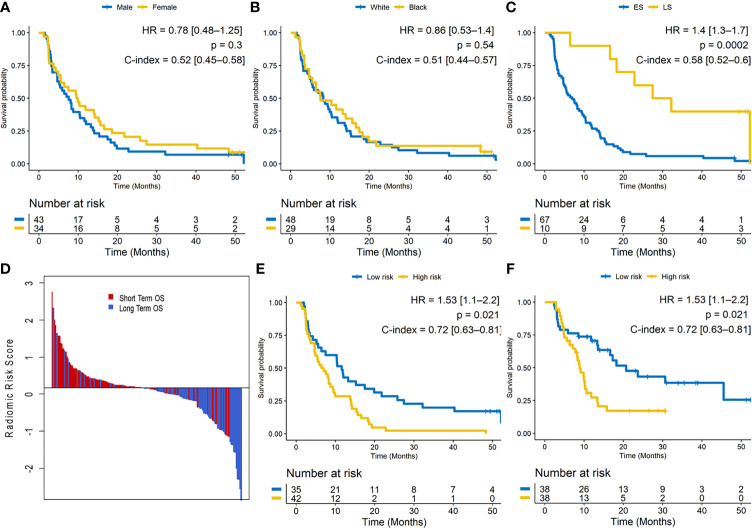
**(A–C)** Kaplan–Meier survival curves for gender, race, and clinical stage on the training set. ES, extensive stage; LS, limited stage. **(D)** Waterfall plot of the length of overall survival (OS) based on radiomic risk score (RRS); higher risk score is associated with lower OS. **(E)** Kaplan–Meier survival curves based on the training set and **(F)** test set. A significant association of the radiomic risk score with the OS is shown in the training set and test set.

The median OS was 8.27 months (IQR = 12.55, [10.10–16.40]). A univariable Cox regression analysis in training set identified that OS was not significantly different for gender (male vs. female; HR: 0.78 [0.48–1.25]; p = 0.3; C-index = 0.52 [0.45–0.58]) or race (HR: 0.86 [0.53–1.4]; p = 0.54; C-index = 0.51 [0.44–0.57]) but was significantly different for clinical stage (LS vs. ES; HR: 1.4 [1.3–1.7]; p = 0.0002; C-index = 0.58 [0.52–0.6]). Especially, patients with brain metastasis demonstrated poorer survival, but in our dataset, this finding was not significantly associated with OS (HR: 0.52; [0.27–0.98]; p = 0.069; C-index = 0.57). **Figures 2A**–**C** illustrate the Kaplan–Meier curves for different clinical factors.

The radiomic score was calculated as a linear combination of the six selected features weighted by their respective coefficients. These features were identified as entropy of intratumoral Haralick feature, median of intratumoral Laws texture feature, peritumoral laws texture feature, intratumoral low-frequency Gabor feature, and peritumoral high-frequency Gabor feature. The optimum cutoff value (the median) for the RRS was found to be 0.17, and patients were stratified into high- and low-risk groups based on this value (**Figure 2D**). A univariable Cox regression analysis developed using textural features indicated that RRS was significantly associated with OS in S^tr^ (HR: 1.53 [1.1–2.2]; p = 0.021; C-index = 0.72 [0.63–0.81]) and S^te^ (HR: 1.4 [1.1–1.82]; p = 0.0127; C-index = 0.69 [0.60–0.77]). The corresponding Kaplan–Meier survival curves showed a significant difference in OS between patients with low and high RRS (p-value <0.05). The Kaplan–Meier survival curves for S^tr^ and S^te^ are shown in **Figures 2E, F**, respectively.

A multivariable Cox regression analysis identified the RRS and cancer staging (limited or extensive stage) as two major risk factors in OS for patients in S^tr^ (RRS: HR = 2.1 [1.53–2.85], p = 0.0076; clinical stage: HR = 1.66 [1.01, 2.7], p = 0.048; race: HR = 0.37 [0.12, 1.1], p = 0.071; and age: HR = 1.04 [0.99–1.09], p = 0.071; C-index = 0.75 [0.68–0.81]) and S^te^ (RRS: HR = 1.9 [1.23–2.2], p = 0.0012; clinical stage: HR = 1.61 [1.2–2.17], p = 0.041; race: HR = 0.86 [0.52–1.42], p = 0.56; and age: HR = 1.01 [0.99–1.03], p = 0.22; C-index = 0.71 [0.64–0.77]).

### 3.3 Integrating Clinical Parameters With Radiomic Features From Pretreatment CT Scans for Prediction of Overall Survival in Small Cell Lung Cancer

**Figure 3:**
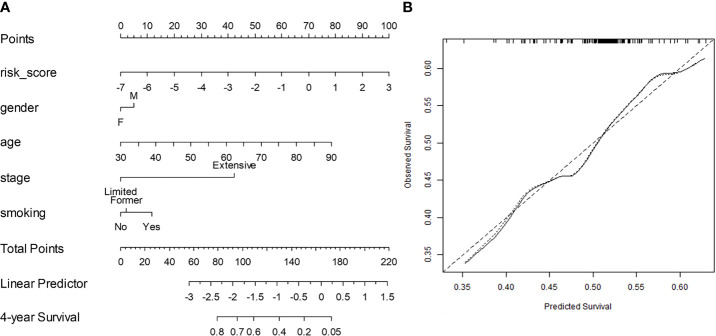
**(A)** Integrated clinical and radiomic nomogram (N^Rad+Clin^) for small cell lung cancer (SCLC) patients treated with systemic chemotherapy estimating the probability of surviving for 4 years. Instructions for reading the nomogram: locate the risk score on the risk score axis. Draw a line straight up to the Points axis to determine how many points toward the predicted probability of a 4-year overall survival (OS) that the patient receives for radiomic risk score level. Repeat this process for the other predictors, each time drawing a line straight up to the Points axis. Sum the points achieved for each predictor and locate this sum on the Total Points axis. Draw a line straight down to the 4-year Survival axis to determine the patient’s probability of surviving for 4 years. Variables with the greatest discriminatory value are those with the widest point range in the nomogram. Sample data from one patient is shown (tan arrows and ovals). **(B)** Calibration curve for 4-year survival. The x-axis shows the nomogram predicted probability, while the y-axis gives the actual 4-year survival as estimated by the Kaplan–Meier method. The dotted line represents an ideal agreement between actual and predicted probabilities of 4-year survival. The solid line represents N^Rad+Clin^ nomogram, and the vertical bars represent 95% CIs. Dots correspond to apparent predictive accuracy.

The C-index for N^Rad+Clin^ to predict OS in S^tr^ was 0.68 [0.66–0.69]. In S^te^, the C-index was 0.67 [0.63–0.68], a value that was greater than that of the conventional clinical-based model (0.54 [0.52–0.56]; p = 0.0024) and RRS alone (0.62 [0.60–0.64]; p = 0.041). The nomogram and the corresponding calibration curve are illustrated in **Figures 3A, B**, respectively. The calibration plot demonstrates an optimal consistency between N^Rad+Clin^ predicted and actual observed OS in S^te^.

**Figure 4:**
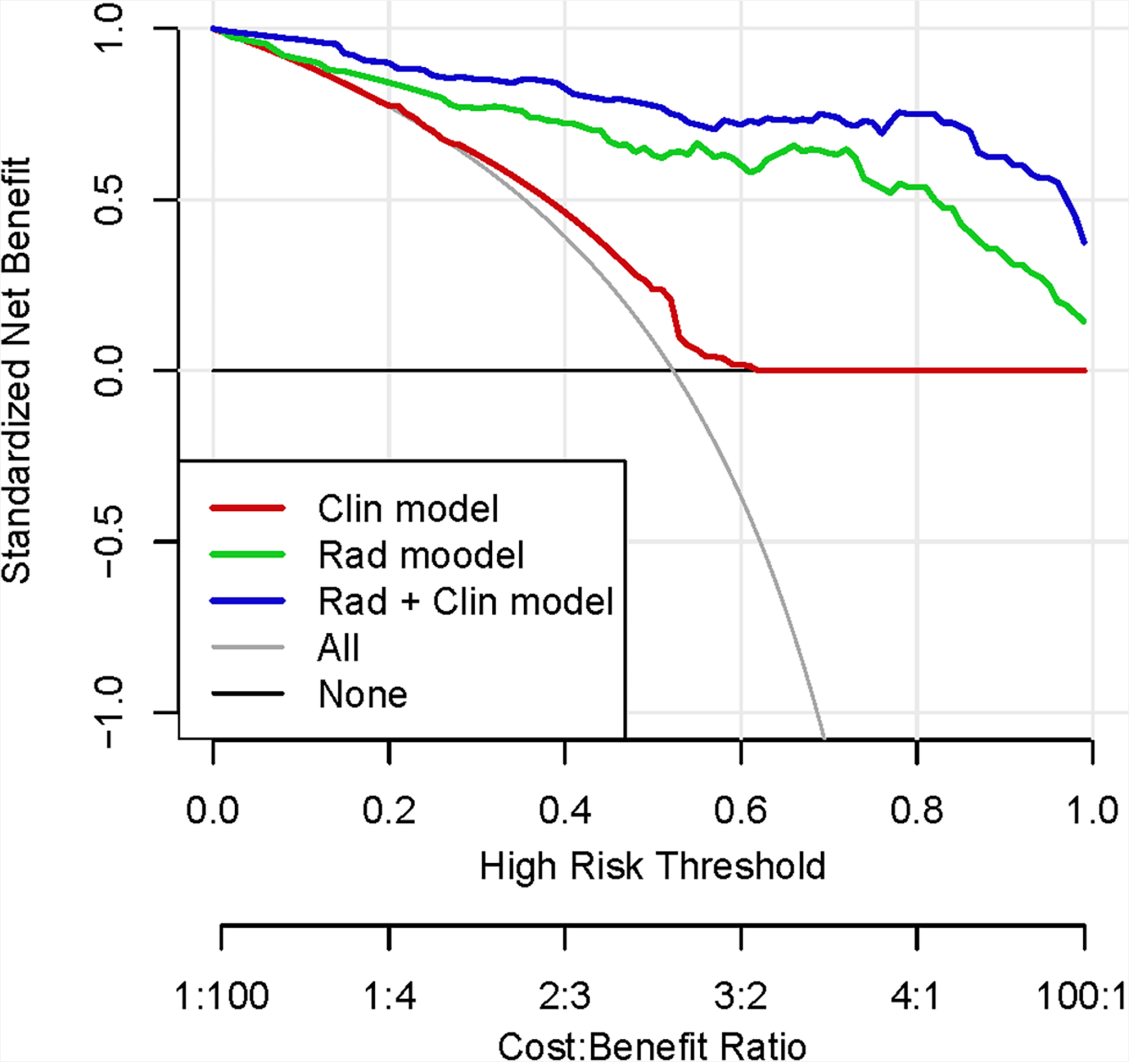
Decision curve analysis (DCA) for each model (clinical model, radiomic model, and integrated Rad+Clin model). The integrated Rad+Clin model had the highest net benefit in predicting which high-risk patients should receive more aggressive treatment, as compared with radiomic model, a clinical model, and simple strategies such as to treat all patients or no patients. This analysis was performed across the full range of threshold probabilities at which a patient would be selected to undergo follow-up imaging.

Moreover, **Figure 4** shows a DCA for three models (clinical model, radiomic model, and integrated Rad+Clin model). As can be seen, the Rad+Clin model had the highest net benefit in predicting which high-risk patients should receive more aggressive treatment as compared with the low-risk patients. The Rad+Clin model yielded a greater net benefit compared with the “treat-all” or “treat-none” strategies.

### 3.4 Radiomic Features From Pretreatment CT Scans Were Associated With Progression-Free Survival in Small Cell Lung Cancer

The median PFS was 7.57 months (IQR = 12.2, [9.68–17.36]). A univariable Cox regression analysis in training set indicated that RRS generated for OS was also significantly associated with PFS in S^tr^ (HR = 1.89 [1.4–4.61], p = 0.047; C-index = 0.7 [0.61–0.78]) and S^te^ (HR = 1.64 [1.1–2.77], p = 0.04; C-index = 0.67 [0.60–0.74]). A multivariable Cox regression analysis identified the RRS and cancer staging as two major risk factors in PFS for patients in S^tr^ (RRS: HR = 1.8 [1.48–2.23], p = 0.0082; clinical stage: HR = 1.35 [1.08, 1.78], p = 0.033; C-index = 0.72 [0.64–0.79]) and S^te^ (RRS: HR = 1.61 [1.13, 1.96], p = 0.0046; clinical stage: HR = 1.37 [1.1, 2.01], p = 0.039; C-index = 0.70 [0.62–0.78]).

### 3.5 Radiomic Features From Pretreatment CT Scans Predict Response to Chemotherapy in Small Cell Lung Cancer

**Figure 5:**
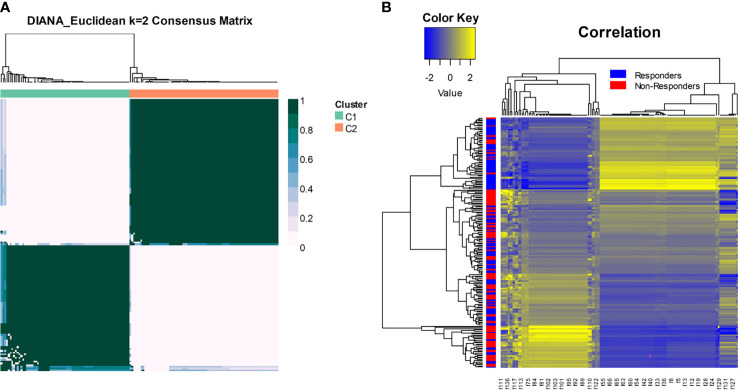
**(A)** Consensus clustering using radiomic features. The two clusters had a preponderance of responders (67%) and non-responders (75%). **(B)** The radiomic heatmap shows an association between the radiomic features and chemotherapy response for patients in the training and test sets.

**Figure 5A** shows distinct response associated clusters obtained *via* consensus clustering performed on a combination of features that were found to be discriminating between responders and non-responders. The two clusters had a preponderance of responders (67%) and non-responders (75%). The radiomic heatmap in **Figure 5B** shows an association between the radiomic features and chemotherapy response for all the patients included in this study.

M^Rad^ was able to predict response to chemotherapy with an AUC of 0.76 [0.75–0.79] within S^tr^ and a corresponding AUC of 0.72, an accuracy of 0.74, a precision of 0.62 (p < 0.05), a specificity of 0.85, a sensitivity of 0.75, and kappa agreement of 0.56 within S^te^.

**Figure 6:**
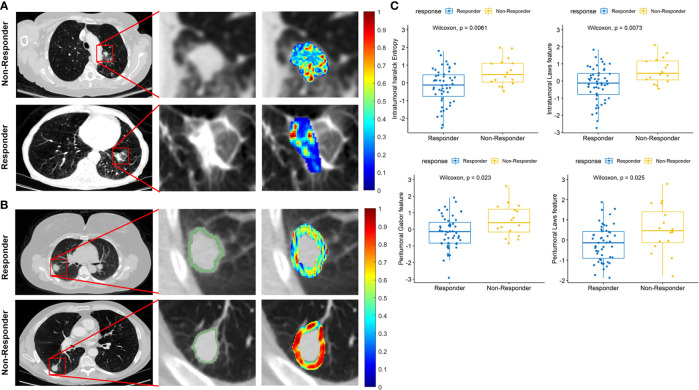
**(A)** Segmented tumor regions and heatmap of intratumoral Haralick (entropy) feature in the pretreatment CT scans for representative non-responder and responder patients. **(B)** Segmented tumor regions and heatmap of peritumoral Gabor feature in the pretreatment CT scans for representative non-responder and responder patients. The middle column is a magnified view of tumor anatomy in both **Figures 5A, B**, and the right column is a color heatmap. **(C)** Box-and-whisker plots for four features that best distinguish chemotherapy response.

**Figures 6A, B** illustrate the discriminability of the intratumoral Haralick entropy and peritumoral Gabor feature for representative non-responder and responder SCLC patients before chemotherapy on the baseline CT scan. There appears to be a higher textural pattern disorder or heterogeneity within and around lesions on CT images before treatment in non-responder patients as compared with responders. This trend is also reflected in the box-and-whisker plots of the Haralick entropy and Gabor texture, illustrated in **Figure 6C**.

## 4 Discussion

SCLC is an aggressive NE tumor of the lung, which arises from bronchial mucosa and shares many morphologic features of NE tumors (26). Chemotherapy remains the backbone of systemic treatment in SCLC. Even though most patients respond to initial treatment, relapse is inevitable, and a subset of patients are chemoresistant. Historically, SCLC was considered as a homogenous disease, and there is now preclinical evidence of inter-tumor heterogeneity with distinct molecular subtypes. Also, recent studies have demonstrated that switching of subtype within the tumor could be a reason for chemotherapeutic resistance (27–30). Currently, there are no clinically validated predictive biomarkers to select a subpopulation of patients with primary chemoresistance and/or early recurrence.

In this study, we presented novel, computer-extracted, quantitative texture features from within and around the tumor lesion from baseline CT scans that are predictive of chemoresistance and prognostic of OS, independent of clinicopathologic factors. Following additional multi-site validation, these non-invasive radiomic biomarkers can be used to predict chemoresistance upfront and orient patients to clinical trials targeting unique therapeutic vulnerabilities to improve clinical outcomes. The primary goal of this study was to determine whether a durable prediction of response to chemotherapy is possible by using radiomic texture patterns within and outside the SCLC tumor on baseline CT scans. We developed a novel RRS derived from computerized texture features from pretreatment CT that was shown to be prognostic of OS and PFS and predictive to chemotherapy response. Additionally, we presented a novel prognostic nomogram (N^Rad+Clin^) that combines radiomic features with clinicopathologic parameters to predict OS.

In this study, we found that the entropy of the intratumoral Haralick feature as well as the median of the intratumoral Laws texture feature had a higher expression in non-responders compared with the responders. These features can capture intratumoral heterogeneity of the tumor (15). High expression of pretreatment serum vascular endothelial growth factor (VEGF) is an important regulator of angiogenesis and vascular permeability in the cell and is known to be associated with poor response to treatment and unfavorable survival in patients with SCLC treated with chemotherapy (31). CD56 is another neural cell adhesion molecule (NCAM) expressed on the cells of tumors of NE origin including SCLC. The overexpression of CD56 is associated with lower OS in SCLC since it inhibits tubulin polymerization and microtubule assembly, causing healthy cell death (32). In addition, increased expression of metalloproteinases (MMPs) is associated with poor prognosis. While we did not explicitly confirm this association, it may be that the overexpression of intratumoral Haralick and Laws features reflect the overexpression of VEGF and CD56 inside a tumor, which in turn are associated with poor OS and response to chemotherapy. Another reason could also be that certain high-risk radiomic features might be reflective of tumor hypoxia. Histological examination of SCLC biopsies showed that at least half of all newly diagnosed SCLC patients have tumor hypoxia (33), which promotes tumor proliferation, increases the metastatic potential, and confers resistance to therapy (34). Also, hypoxic regions of the tumor are relatively devoid of blood vessels, making it difficult for drugs to diffuse and reach to the tumor bed.

Our experiment showed that the peritumoral Laws texture feature had higher expression in non-responders compared with the responders and associated with lower OS. Our findings could provide new insight into tumor microenvironment biology, while its appearance on radiographic imaging may be explained as follows. SCLC can be classified into NE‐high and NE‐low tumors, based on a different immunogenic expression. NE‐high is defined as a cold or “immune desert” phenotype, based on low levels of immune cell expression, whereas NE‐low is defined as a “hot” or “immune oasis” phenotype associated with increased immunogenicity (35, 36). This extrapolates to better therapy response in NE‐low SCLC as compared with NE-high SCLC patients (37, 38). The differences observed in the expression of peritumoral Laws texture features between responders and non-responders could be related to differential expression of immune cells around the tumor.

Our result showed that the RRS and cancer staging (limited or extensive stage) were only two major risk factors in predicting OS in both training (RRS: HR = 2.1, [1.53, 2.85], p = 0.0076; clinical stage: HR = 1.66, [1.01, 2.7], p = 0.048; race: HR = 0.37, [0.12, 1.1], p = 0.071; and age: HR = 1.04, [0.99, 1.09], p = 0.071; C-index = 0.75) and test sets (RRS: HR = 1.9, [1.23, 2.2], p = 0.0012; clinical stage: HR = 1.61, [1.2, 2.17], p = 0.041; race: HR, 0.86, 95% CI: [0.52, 1.42], p = 0.56; and age: HR, 1.01, 95% CI: [0.99, 1.03], p = 0.22; C-index = 0.71). Moreover, RRS and cancer staging were also two major risk factors in predicting PFS in training (RRS: HR = 1.8, [1.48, 2.23], p = 0.0082; clinical stage: HR = 1.35, [1.08, 1.78], p = 0.033; C-index = 0.72) and test sets (RRS: HR = 1.61, [1.13, 1.96], p = 0.0046; clinical stage: HR = 1.37, [1.1, 2.01], p = 0.039; C-index = 0.70). In addition, while radiomic features were associated with response to chemotherapy, the clinical biomarkers (age, sex, and tumor stage) were not able to predict response to chemotherapy.

Finally, we presented a nomogram that integrated RRS with clinical biomarkers (N^Rad+Clin^) to further improve its prognostic accuracy. The N^Rad+Clin^ exhibited the highest C-index value in both training and test cohorts as compared with the clinical or radiomic models alone. We also evaluated N^Rad+Clin^ by DCA and calculated the net benefit of our model. The decision curve indicated that N^Rad+Clin^ had the highest overall net benefit in predicting high-risk patients for receiving more aggressive treatment than the clinicopathologic measurements across all threshold probability values. With the capability to assess prognosis and response to therapy upfront, the oncologist can be assisted with decision making to estimate therapeutic outcomes for a given patient to reduce ineffective treatments and/or associated toxicity.

To the best of our knowledge, this study is the first to explore the relationship of radiomic features with chemotherapy response and OS in SCLC patients. The convergence of these areas provides a new radiomic model that could yield effective non-invasive prediction of treatment response without sacrificing transparency of biological rationale.

We acknowledge that our study did have its limitations. The cohort sizes for both training and test are relatively small; however, given the relatively low incidence of SCLC as compared with other lung cancer subtypes, the number of patients in the historic studies has been small as well. We also studied a single institution, which may affect the results. Additionally, a couple of recent studies have rigorously and quantitatively investigated the influence of convolution kernels, reconstruction algorithms, and slice thickness on radiomic features for characterization of lung nodules on CT. In the present study, we did not explicitly consider the influence of these parameters on the extracted texture features but randomly distributed the cases with different image acquisition parameters between training and test sets to account for variability. Since features pertaining to radiographic images reflect hallmarks of tumor biology and not drug/agent specific, we expect that the predictive aspects of these features will remain broadly similar across different chemotherapeutic agents. We hope to address in future studies the rigorous morphologic and molecular underpinning of the radiomic findings that were shown in this study.

## 5 Conclusion

In conclusion, our results suggest that radiomic texture features from baseline CT scans of SCLC patients can predict resistance to platinum-based chemotherapy. Our study highlights that these radiomic features are also associated with OS in patients with SCLC, and we presented an integrated nomogram that can estimate the survival probability based on the RRS and clinical biomarkers.

## Data Availability Statement

Data are available upon reasonable request. Access to datasets from the University Hospitals Cleveland Medical Center (used with permission for this study) should be requested directly from these institutions via their data access request forms. Subject to the institutional review boards’ ethical approval, unidentified data would be made available as a test subset.

## Ethics Statement

This study was conducted in full accordance with the Health Insurance Portability and Accountability Act (HIPAA) regulations after approval from the Institutional Review Board at Case Western Reserve University (Cleveland, OH), and the IRB waived the requirements for informed consent of all patients because of the retrospective, non-interventional, and non-therapeutic nature of this study.

## Author Contributions

Conceptualization: PJ, MK, and AM. Methodology: PJ, MK, and AM. Software: MK. Validation: PJ, MK, and AM. Formal analysis: MK, PJ, and PF. Investigation: MK and PJ. Resources: MK. Data curation: PJ, KB, PR, AG, VV, and AD. Writing—original draft preparation: PJ, MK, and AG. Writing—review and editing: PJ, MK, AG, KB, PR, VV, PF, AD, and AM. Visualization: MK. Supervision: AM. All authors contributed to the article and approved the submitted version.

## Funding

The research reported in this publication was supported by the National Cancer Institute under award numbers 1U24CA199374-01, R01CA202752-01A1, R01CA208236-01A1, R01CA216579-01A1, R01CA220581-01A1, 1U01CA239055-01, 1U01CA248226-01, and 1U54CA254566-01, National Heart, Lung and Blood Institute 1R01HL15127701A1, National Institute for Biomedical Imaging and Bioengineering 1R43EB028736-01, National Center for Research Resources under award number 1 C06 RR12463-01, VA Merit Review Award IBX004121A from the United States Department of Veterans Affairs Biomedical Laboratory Research and Development Service, the Office of the Assistant Secretary of Defense for Health Affairs, through the Breast Cancer Research Program (W81XWH-19-1-0668), the Prostate Cancer Research Program (W81XWH-15-1-0558, W81XWH-20-1-0851), the Lung Cancer Research Program (W81XWH-18-1-0440, W81XWH-20-1-0595), the Peer Reviewed Cancer Research Program (W81XWH-18-1-0404), the Kidney Precision Medicine Project (KPMP) Glue Grant, the Ohio Third Frontier Technology Validation Fund, the Clinical and Translational Science Collaborative of Cleveland (UL1TR0002548) from the National Center for Advancing Translational Sciences (NCATS) component of the National Institutes of Health and NIH roadmap for Medical Research, and The Wallace H. Coulter Foundation Program in the Department of Biomedical Engineering at Case Western Reserve University. The content is solely the responsibility of the authors and does not necessarily represent the official views of the National Institutes of Health, the U.S. Department of Veterans Affairs, the Department of Defense, or the United States Government.

## Conflict of Interest

AM is an equity holder in Elucid Bioimaging and in Inspirata, Inc. In addition, he has served as a scientific advisory board member for Inspirata, Inc., AstraZeneca, Bristol Meyers-Squibb, and Merck. Currently, he serves on the advisory board of Aiforia Inc. He also has sponsored research agreements with Philips, AstraZeneca, Boehringer-Ingelheim, and Bristol Meyers-Squibb. His technology has been licensed to Elucid Bioimaging. He is also involved in a NIH U24 grant with PathCore Inc. and three different R01 grants with Inspirata, Inc. AD has received consultancy fees for advisory committees from BMS, AZ, Bayer, and Jazz Pharmaceuticals. AG has received research support from General Electric Healthcare.

The remaining authors declare that the research was conducted in the absence of any commercial or financial relationships that could be construed as a potential conflict of interest.

## Publisher’s Note

All claims expressed in this article are solely those of the authors and do not necessarily represent those of their affiliated organizations, or those of the publisher, the editors and the reviewers. Any product that may be evaluated in this article, or claim that may be made by its manufacturer, is not guaranteed or endorsed by the publisher.

## References

[1] Lemjabbar-Alaoui H Hassan OU Yang YW Buchanan P. Lung Cancer: Biology and Treatment Options. Biochim Biophys Acta (2015) 1856(2):189–210. doi: 10.1016/j.bbcan.2015.08.002 26297204 PMC4663145

[2] Alvarado-Luna G Morales-Espinosa D. Treatment for Small Cell Lung Cancer, Where Are We Now?-A Review. Transl Lung Cancer Res (2016) 5(1):26–38. doi: 10.3978/j.issn.2218-6751.2016.01.13 26958491 PMC4758961

[3] Riaz SP Lüchtenborg M Coupland VH Spicer J Peake MD Møller H. Lung Cancer - Small Cell: Statistics [Webpage on the Internet]. Alexandria, VA: American Society of Clinical Oncology (ASCO; Cancer.Net (2019). Available at: https://www.cancer.net/cancer-types/lung-cancer-small-cell/statistics.

[4] Hurwitz JL McCoy F Scullin P Fennell DA. New Advances in the Second-Line Treatment of Small Cell Lung Cancer. Oncologist (2009) 14(10):986–94. doi: 10.1634/theoncologist.2009-0026 19819917

[5] Schneider BJ. Management of Recurrent Small Cell Lung Cancer. J Natl Compr Canc Netw (2008) 6(3):323–31. doi: 10.6004/jnccn.2008.0027 18377850

[6] Tas F Ciftci R Kilic L Karabulut S. Age Is a Prognostic Factor Affecting Survival in Lung Cancer Patients. Oncol Lett (2013) 6(5):1507–13. doi: 10.3892/ol.2013.1566 PMC3813578 24179550

[7] Fischer BM Mortensen J Langer SW Loft A Berthelsen AK Petersen BI. A Prospective Study of PET/CT in Initial Staging of Small-Cell Lung Cancer: Comparison With CT, Bone Scintigraphy and Bone Marrow Analysis. Ann Oncol (2007) 18(2):338–45. doi: 10.1093/annonc/mdl374 17060487

[8] Sandler AB. Current Management of Small Cell Lung Cancer. Semin Oncol (1997) 24(4):463–76. doi: 10.1016/j.ccm.2011.07.002 9280226

[9] Kalemkerian GP Akerley W Bogner P Borghaei H Chow LQ Downey RJ. National Comprehensive Cancer Network. Small Cell Lung Cancer. J Natl Compr Canc Netw (2013) 11(1):78–98. doi: 10.6004/jnccn.2013.0011 23307984 PMC3715060

[10] Foster NR Mandrekar SJ Schild SE Nelson GD Rowland KM Jr Deming RL. Prognostic Factors Differ by Tumor Stage for Small Cell Lung Cancer: A Pooled Analysis of North Central Cancer Treatment Group Trials. Cancer (2009) 115(12):2721–31. doi: 10.1002/cncr.24314 PMC2779694 19402175

[11] Dowlati A Lipka MB McColl K Dabir S Behtaj M Kresak A. Clinical Correlation of Extensive-Stage Small-Cell Lung Cancer Genomics. Ann Oncol (2016) 27(4):642–7. doi: 10.1093/annonc/mdw005 PMC4803453 26802149

[12] Gillies RJ Kinahan PE Hricak H. Radiomics: Images Are More Than Pictures, They Are Data. Radiology (2016) 278(2):563–77. doi: 10.1148/radiol.2015151169 PMC4734157 26579733

[13] Khorrami M Prasanna P Gupta A Patil P Velu PD Thawani R. Changes in CT Radiomic Features Associated With Lymphocyte Distribution Predict Overall Survival and Response to Immunotherapy in Non-Small Cell Lung Cancer. Cancer Immunol Res (2020) 8(1):108–19. doi: 10.1158/2326-6066.CIR-19-0476 PMC7718609 31719058

[14] Khorrami M Khunger M Zagouras A Patil P Thawani R Bera K. Combination of Peri- and Intratumoral Radiomic Features on Baseline CT Scans Predicts Response to Chemotherapy in Lung Adenocarcinoma. Radiol Artif Intell (2019) 1(2):e180012. doi: 10.1148/ryai.2019180012 32076657 PMC6515986

[15] Khorrami M Jain P Bera K Alilou M Thawani R Patil P. Predicting Pathologic Response to Neoadjuvant Chemoradiation in Resectable Stage III Non-Small Cell Lung Cancer Patients Using Computed Tomography Radiomic Features. Lung Cancer (2019) 135:1–9. doi: 10.1016/j.lungcan.2019.06.020 31446979 PMC6711393

[16] Vaidya P Bera K Patil PD Gupta A Jain P Alilou M. Novel, Non-Invasive Imaging Approach to Identify Patients With Advanced Non-Small Cell Lung Cancer at Risk of Hyperprogressive Disease With Immune Checkpoint Blockade. J Immunother Cancer (2020) 8(2):e001343. doi: 10.1136/jitc-2020-001343 33051342 PMC7555103

[17] Budai BK Tóth A Borsos P Frank VG Shariati S Fejér B. Three-Dimensional CT Texture Analysis of Anatomic Liver Segments can Differentiate Between Low-Grade and High-Grade Fibrosis. BMC Med Imaging (2020) 20(1):108. doi: 10.1186/s12880-020-00508-w 32957949 PMC7507285

[18] Khorrami M Bera K Thawani R Rajiah P Gupta A Fu P. Distinguishing Granulomas From Adenocarcinomas by Integrating Stable and Discriminating Radiomic Features on Non-Contrast Computed Tomography Scans. Eur J Cancer (2021) 148:146–58. doi: 10.1016/j.ejca.2021.02.008 PMC8087632 33743483

[19] Eisenhauer EA Therasse P Bogaerts J Schwartz LH Sargent D Ford R. New Response Evaluation Criteria in Solid Tumours: Revised RECIST Guideline (Version 1.1). Eur J Cancer (2009) 45(2):228–47. doi: 10.1016/j.ejca 19097774

[20] Komatsu T Kunieda E Oizumi Y Tamai Y Akiba T. Clinical Characteristics of Brain Metastases From Lung Cancer According to Histological Type: Pretreatment Evaluation and Survival Following Whole-Brain Radiotherapy. Mol Clin Oncol (2013) 1(4):692–8. doi: 10.3892/mco.2013.130 PMC3915483 24649230

[21] Apte AP Iyer A Crispin-Ortuzar M Pandya R van Dijk LV Spezi E. Technical Note: Extension of CERR for Computational Radiomics: A Comprehensive MATLAB Platform for Reproducible Radiomics Research. Med Phys (2018) 13:10.1002/mp.13046. doi: 10.1002/mp.13046 PMC6597320 29896896

[22] Griethuysen JJM Fedorov A Parmar C Hosny A Aucoin N Narayan V. Computational Radiomics System to Decode the Radiographic Phenotype. Cancer Res (2017) 77(21):e104–7. doi: 10.1158/0008-5472.CAN-17-0339 PMC5672828 29092951

[23] Armato SG 3rd Meyer CR Mcnitt-Gray MF McLennan G Reeves AP Croft BY. The Reference Image Database to Evaluate Response to Therapy in Lung Cancer (Rider) Project: A Resource for the Development of Change-Analysis Software. Clin Pharmacol Ther (2008) 84:448–56. doi: 10.1038/clpt.2008.161 PMC4938843 18754000

[24] Vickers AJ Elkin EB. Decision Curve Analysis: A Novel Method for Evaluating Prediction Models. Med Decis Making (2006) 26(6):565–74. doi: 10.1177/0272989X06295361 PMC2577036 17099194

[25] Li W Nyholt DR. Marker Selection by Akaike Information Criterion and Bayesian Information Criterion. Genet Epidemiol (2001) 21(Suppl 1):S272–7. doi: 10.1002/gepi.2001.21.s1.s272 11793681

[26] Chong S Lee KS Chung MJ Han J Kwon OJ Kim TS. Neuroendocrine Tumors of the Lung: Clinical, Pathologic, and Imaging Findings. Radiographics (2006) 26(1):41–57; discussion 57–8. doi: 10.1148/rg.261055057 16418242

[27] Stewart CA Gay CM Xi Y Sivajothi S Sivakamasundari V Fujimoto J. Single-Cell Analyses Reveal Increased Intratumoral Heterogeneity After the Onset of Therapy Resistance in Small-Cell Lung Cancer. Nat Cancer (2020) 1:423–36. doi: 10.1038/s43018-019-0020-z PMC7842382 33521652

[28] Simpson KL Stoney R Frese KK Simms N Rowe W Pearce SP. A Biobank of Small Cell Lung Cancer CDX Models Elucidates Inter- and Intratumoral Phenotypic Heterogeneity. Nat Cancer (2020) 1:437–51. doi: 10.1038/s43018-020-0046-2 35121965

[29] Ireland AS Micinski AM Kastner DW Guo B Wait SJ Spainhower KB. MYC Drives Temporal Evolution of Small Cell Lung Cancer Subtypes by Reprogramming Neuroendocrine Fate. Cancer Cell (2020) 38(1):60–78.e12. doi: 10.1016/j.ccell.2020.05.001 32473656 PMC7393942

[30] Gay CM Stewart CA Park EM Diao L Groves SM Heeke S. Patterns of Transcription Factor Programs and Immune Pathway Activation Define Four Major Subtypes of SCLC With Distinct Therapeutic Vulnerabilities. Cancer Cell (2021) 39(3):346–60.e7. doi: 10.1016/j.ccell.2020.12.014 PMC8143037 33482121

[31] Salven P Ruotsalainen T Mattson K Joensuu H. High Pre-Treatment Serum Level of Vascular Endothelial Growth Factor (VEGF) Is Associated With Poor Outcome in Small-Cell Lung Cancer. Int J Cancer (1998) 79(2):144–6. doi: 10.1002/(sici)1097-0215(19980417)79:2<144::aid-ijc8>3.0.co;2-t 9583728

[32] Karim SM Zekri J. Chemotherapy for Small Cell Lung Cancer: A Comprehensive Review. Oncol Rev (2012) 6(1):e4. doi: 10.4081/oncol.2012.e4 25992206 PMC4419639

[33] Ioannou M Papamichali R Kouvaras E Mylonis I Vageli D Kerenidou T. Hypoxia Inducible Factor-1 Alpha and Vascular Endothelial Growth Factor in Biopsies of Small Cell Lung Carcinoma. Lung (2009) 187(5):321–9. doi: 10.1007/s00408-009-9169-z 19707816

[34] Bryant JL Meredith SL Williams KJ White A. Targeting Hypoxia in the Treatment of Small Cell Lung Cancer. Lung Cancer (2014) 86(2):126–32. doi: 10.1016/j.lungcan.2014.08.003 25201720

[35] Rudin CM Poirier JT Byers LA Dive C Dowlati A George J. Molecular Subtypes of Small Cell Lung Cancer: A Synthesis of Human and Mouse Model Data. Nat Rev Cancer (2019) 19(5):289–97. doi: 10.1038/s41568-019-0133-9 PMC6538259 30926931

[36] McColl K Wildey G Sakre N Lipka MB Behtaj M Kresak A. Reciprocal Expression of INSM1 and YAP1 Defines Subgroups in Small Cell Lung Cancer. Oncotarget (2017) 8:73745–56. doi: 10.18632/oncotarget.20572 PMC5650296 29088741

[37] Schwendenwein A Megyesfalvi Z Barany N Valko Z Bugyik E Lang C. Molecular Profiles of Small Cell Lung Cancer Subtypes: Therapeutic Implications. Mol Ther Oncolytics (2021) 20:470–83. doi: 10.1016/j.omto.2021.02.004 PMC7917449 33718595

[38] Saito M Shiraishi K Goto A Suzuki H Kohno T Kono K. Development of Targeted Therapy and Immunotherapy for Treatment of Small Cell Lung Cancer. Jpn J Clin Oncol (2018) 48(7):603–8. doi: 10.1093/jjco/hyy068 29762727

